# A Short Patient-Reported Outcome Measure for Oral Anticancer Agents: Multicenter Observational Study

**DOI:** 10.2196/85201

**Published:** 2026-02-18

**Authors:** Emily Mackler, Taylor Weis, Kelly Romo, Michelle Azar, Jennifer J Griggs, Vincent D Marshall, Karen Farris

**Affiliations:** 1 College of Pharmacy, University of Michigan, 1007 E Huron St., Ann Arbor, MI, 48104-1628, United States, 1 7347635150; 2 Michigan Oncology Quality Consortium, Medical School, University of Michigan, Ann Arbor, MI, United States; 3 Medical School Department of Internal Medicine, Hematology and Oncology Division, University of Michigan Medical School, University of Michigan, Ann Arbor, MI, United States

**Keywords:** patient-reported outcomes, symptoms, adverse effects, medication adherence, oral anticancer agent

## Abstract

**Background:**

The Michigan Oncology Quality Consortium developed a rapid patient-reported outcome measure (RapidPRO) focused on oral anticancer agents (OAAs). We piloted this measure in 6 oncology practices to determine its usefulness in representing the symptom experience and medication adherence among individuals taking OAAs. It is common in oncology for cancer-specific approaches to be used. We sought to use 1 instrument for all OAAs as a means to simplify future implementation in practice.

**Objective:**

This study aimed to describe the use of RapidPRO in practice and quantify clinical metrics in RapidPRO for symptom burden, confidence to manage symptoms, confidence to know when to seek care, and OAA medication adherence.

**Methods:**

This observational study was conducted across 6 practices from July 2016 to December 2018. RapidPRO assesses symptoms, patient confidence, and medication adherence with respect to OAAs.

**Results:**

There were 2252 RapidPROs completed by 695 patients. Among individuals completing at least 2 RapidPROs, the median number of days between them was 28 (IQR 14-42). Of the 2252 completed RapidPROs, 1213 (53.9%) reported at least one moderate or severe symptom, and 28% (485/1705) reported medication nonadherence. Most bothersome symptoms (MBSs; n=1045) were reported in 35.1% (790/2252) of the RapidPROs, and 46.5% (323/695) of all patients reported an MBS. In exploratory analyses, RapidPROs that reported a moderate or severe symptom or lower confidence to manage symptoms were more likely to be nonadherent to OAA therapy. The most common reason for medication nonadherence was “experienced side effects.”

**Conclusions:**

These results show that most RapidPROs reported at least one moderate or severe symptom and 28% (485/1705) reported medication nonadherence. As well, RapidPRO was able to capture most patients’ MBSs. By implementing RapidPRO, practices can identify patients who experience symptoms, as well as those who report medication nonadherence.

## Introduction

The increasing use of oral anticancer agents (OAAs) has led to more convenient medication administration and improved patient survival rates [1-3]. OAAs are a heterogeneous group of oral therapies, including cytotoxic chemotherapies, hormonal therapies, targeted therapies, and immunomodulatory agents, that are administered at home under patient self-management, unlike parenteral chemotherapy administered under direct clinical supervision [3-5]. The benefits of OAAs are significant, including receipt of treatment at home, opportunities for patients to carry on with daily activities, and avoidance of complications from intravenous chemotherapy. As well, patients generally prefer oral medication compared to infusions [4-8]. Despite the ease of OAA administration, this shift in responsibility from oncologists and oncology professionals to patients introduces unique challenges that can negatively impact patients’ quality of life and disease course [9], including difficulties related to medication adherence, self-management of toxicities or adverse effects, and misconceptions that OAAs are less effective than intravenous chemotherapy [8,10-15]. A total of 30% of patients have reported that their symptoms are the primary reason for nonadherence to their OAAs [16].

While the use of OAAs has continued to grow, the ways in which practices assess and support medication adherence and self-management of toxicities are inconsistent. However, OAA self-management is crucial and necessitates patient education and confidence to take medication and identify, manage, and/or report treatment side effects [17-21]. Patient-reported outcome measures (PROMs) are reports provided by patients about their own quality of life or functional status as a result of their disease and health care [22]. PROMs provide direct insight into the patient’s perspective on symptoms and treatment-related experiences, complementing clinician assessments [23]. In this study, a rapid PROM tool (RapidPRO) was used as a practical monitoring tool to identify symptom burden and medication adherence concerns in real time [24]. PROMs for symptom monitoring have been found to increase quality of life and overall survival when the information is acted upon by oncologists and oncology professionals [25,26]. Because OAAs require significant self-monitoring and patient adherence as compared to infusion therapies, patients’ confidence to self-monitor and know when to report symptoms is particularly critical. Evidence from cancer populations indicates that greater self-efficacy or confidence in managing treatment and symptoms correlates with better symptom control and higher medication adherence [27-29]. Therefore, assessing patients’ confidence alongside symptom burden may offer a powerful approach to identifying patients at risk of medication nonadherence. While remote symptom-monitoring systems have been increasingly adopted in oncology, assessing patients’ confidence to self-manage proactively as part of a PROM is novel.

The Michigan Oncology Quality Consortium (MOQC) is a continuous quality improvement collaborative comprising 46 ambulatory oncology practices that collect and share data about the quality of cancer care. They also implement changes, seeking to improve oncology care and outcomes across the state of Michigan [26-28]. The MOQC developed a PROM (RapidPRO), and it was distributed at selected practice sites to monitor symptom burden and medication adherence. Having 1 PROM rather than drug- or condition-specific measures is likely to increase uptake of PROMs because of the simplicity of using 1 measure and understanding how to use and interpret it [26,29]. Additionally, prior evidence shows that using a single standardized PROM across diverse cancer populations enhances feasibility, supports consistent quality improvement efforts, and provides actionable patient-reported data in routine oncology practice [30]. Therefore, the objective of this study was to (1) describe the use of the RapidPRO in oncology practice and (2) quantify the clinical metrics captured in the RapidPRO for symptom burden, confidence to manage symptoms, confidence to know when to seek care, and medication adherence. In routine practice, it was unclear whether 1 short PROM could be used in daily oncology practice to assess the use of OAAs. What is challenging is working with oncology practices to incorporate such assessments routinely into their workflows, especially before visits so that oncologists and oncology professionals can then discuss the findings with patients during visits. These preliminary results can help us understand whether symptomatology is prevalent and complex across many OAAs and whether such assessments could increase the benefits of cancer care delivery.

## Methods

### Study Design

This archival, multicenter, observational quality improvement study was conducted across 6 practice sites in the state of Michigan from July 2016 to December 2018. A total of 2252 PROMs were completed by 695 patients from 6 practices during the study period. Patients were eligible to be included in the study if they were aged 18 years or older and taking a US Food and Drug Administration–approved OAA for the treatment of cancer (excluding endocrine therapy). A focus on OAAs excluding endocrine therapy was chosen to emphasize medications with more acute toxicities for which additional support from clinic staff for medication adherence and/or symptom management may be needed. Patients completing the RapidPRO while on OAA therapy were included, and patients could complete more than one RapidPRO during their treatment. Patients completing the assessments could receive concurrent intravenous chemotherapy in addition to an OAA. Six practices participated in this quality improvement study. The practices ranged in size from approximately 400 to 3000 new oncology patients per year and from 2 to 12 full-time medical hematology and oncology physicians. They were geographically located across Michigan and were not academic medical centers.

### Ethical Considerations

This study was reviewed by the University of Michigan Institutional Review Board and was determined to be not regulated (nonregulated studies do not require informed consent) as human subject research because it met institutional criteria for quality improvement activities (HUM00099930). The MOQC reimbursed each site a stipend of US $2000 as compensation for their effort in deidentifying the PROM data and sending them to the MOQC. Payment occurred at the completion of 12 months of participation.

### Data Collection Process

MOQC practices were able to volunteer to participate in this phase of the MOQC Oral Oncolytics program after they learned about it at the MOQC Biannual Meeting. Each participating practice took part in an introduction phone call where they discussed their current process for managing patients receiving OAAs. All practices also learned about the following five requirements for participation: (1) completion of a baseline survey to quantify the perception of OAA nationally recognized standards; (2) completion of a baseline chart audit (20 patients on OAAs) to quantify OAA nationally recognized standards in the practice; (3) implementation of the RapidPRO at the site for tracking patients’ symptoms and medication adherence, where practices were instructed to have patients complete the RapidPRO once within the first month of therapy (ideally 7-10 days after initiation) and then routinely at each outpatient visit; (4) collection of at least 12 months of PROM data sent to the MOQC for evaluation; and (5) completion of a postimplementation survey related to OAA nationally recognized standards. Key team members from each practice also participated in an onboarding call with the MOQC coordinating team. During this call, the MOQC provided training on the use of PROMs for symptom follow-up in oncology, the importance of medication adherence during OAA therapy, and best practices from early pilot sites. Practices were also given a concise user guide with recommended follow-up actions based on symptom thresholds and instructions for integrating RapidPRO into workflows. A standardized onboarding process and a single MOQC user guide were provided to all sites to promote uniform administration. To further ensure consistency, all completed RapidPRO forms were centrally entered into REDCap (Research Electronic Data Capture; Vanderbilt University) by MOQC staff rather than by individual sites.

Practices were expected to participate in regular phone calls with frequency determined at the discretion of the MOQC team and the oncology practice. On-site meetings with the MOQC team and MOQC support in developing new workflows were offered and took place on an as-needed basis. Practices were offered a tablet computer if they wished to use it for patients to complete the RapidPRO at the office, and a RapidPRO paper version was also available.

### RapidPRO Measure

The RapidPRO was developed to examine symptom burden, confidence in self-managing side effects, and adherence to OAAs [27,31,32]. RapidPRO is a 19-item, 2-page survey that takes approximately 3 minutes to complete. Each patient completed 1 RapidPRO per assessment period regardless of the number of OAAs prescribed. The measure was designed to capture overall treatment-related symptoms and medication adherence without attributing specific symptoms to individual medications. It incorporates a modified version of the Edmonton Symptom Assessment System (ESAS), a validated tool originally designed to assess general symptom burden among patients with cancer [33]. The ESAS has been modified in several contexts to address symptom monitoring in specific populations, such as outpatients with advanced cancer [34]. The modified ESAS in the RapidPRO collected patients’ symptom severity on a scale from 0 to 10 (0=none; 10=worst possible). The literature indicates that the ESAS has validity and reliability for patients with cancer [34,35]. For the classification of symptom burden, a score of 1 to 3 was considered mild, a score of 4 to 6 was considered moderate, and a score of 7 to 10 was considered severe [27], and these severity thresholds are consistent with established scoring conventions used in the ESAS literature [33,36]. Symptoms assessed included pain, tiredness, drowsiness, nausea, lack of appetite, shortness of breath, depression, anxiety, best well-being, constipation, diarrhea, tingling or numbness, mouth sores, and rash.

Additionally, a question in the PROM asked patients in a free-text format to identify the most bothersome symptom (MBS) they were experiencing, and patients were allowed to list multiple MBSs. During analysis, 3 clinical reviewers (a medical oncologist, a nurse, and a pharmacist) independently reviewed the MBS responses to categorize them by symptom type based on the review of systems classification [37]. If the categorization for a symptom was inconsistent between reviewers, the symptom was re-evaluated to reach a consensus.

The PROM also included questions asking patients to rate their confidence in managing side effects and their confidence in seeking medical care for side effects on a scale from 0 to 10 (0=“I’m not confident”; 10=“I am confident”) [27,31,32].

Next, patients were asked to rate their medication adherence over the previous month using a 5-point scale (“excellent,” “very good,” “good,” “fair,” or “poor”). This single-item measure, previously validated in studies of patients taking antiretroviral therapy, demonstrated good concordance with objective medication adherence data based on pill bottle openings. The 1-month recall period has been shown to reduce overreporting compared with 3- and 7-day recall windows. Similarly, using a 5-point rating scale rather than frequency-based measures further minimized overreporting of medication adherence [38,39], and less than “excellent” corresponds approximately to <80% adherence [38,39]. Because routine assessment of medication adherence in daily oncology practice is challenging, this single-item measure provides a practical approach to identifying patients at risk of medication nonadherence. Reasons for not taking one’s OAAs, such as experiencing side effects, financial issues, and forgetfulness, were also assessed [27,31]. Conceptually, the purpose of RapidPRO is to provide an opportunity for patients to report their symptoms and medication experiences outside the office visit and for oncologists and oncology professionals and personnel to follow up proactively on those reports when needed.

Additional information and links to the complete RapidPRO intake form, assessment forms, and tool guide are available in Multimedia Appendix 1.

### Data Analysis

Practices submitted completed, deidentified RapidPRO forms to the MOQC for entry into REDCap, which is a password-protected, user-authenticated, encrypted, and firewalled application used to collect and enter sensitive data in compliance with HIPAA (Health Insurance Portability and Accountability Act). Standard audits took place throughout data entry. Discrepancies were resolved by the project manager. Missing data were accounted for by presentation of the valid sample size, with noninclusion in the denominator. The regression had missing values that were removed listwise.

For objective 1, practice-level metrics were established, including the number of PROMs completed per week and the mode of administration. The average, median number, and range of PROMs per medication, as well as by days between PROMs, were determined and reported for medications with at least 100 PROMs. The number of PROMs, as well as the number of patients represented by those PROMs, was determined.

For objective 2, the patient-reported symptoms and medication adherence were quantified using frequency distributions. This analysis aimed to evaluate whether changes in symptom burden and confidence to manage or seek care were associated with medication adherence over time. Interaction terms (time × symptom burden and time × confidence) were included to test whether these associations changed across repeated assessments in individuals with 2 or more PROMs.

We used a predictive generalized linear mixed interaction model as well as smoothing splines for presentation of results. A random intercept for each patient ID allowed for handling of intrapatient correlation. All data were analyzed using R (version 3.5.2; R Foundation for Statistical Computing) [40,41], and the generalized linear mixed model was performed using the R package *lme4* [42].

## Results

### RapidPRO Data Collection

The number of PROMs completed per week varied by practice (Table 1), and all practices preferred paper forms for completion. Almost one-third of PROMs (710/2252, 31.5%) were completed by patients as part of a self-assessment during visits, and at least 2 practices had the PROM completed during a telephone follow-up by a pharmacist or nurse.

Across practices, substantial variation in clinic size and staffing was observed, including annual new oncology patient volume ranging from 421 to 3000 and the number of oncologists and oncology professionals ranging from 2 to 12 per site (Table 1). These differences were reflected in RapidPRO implementation metrics, with PROM completion per site ranging from 79 to 1411 assessments over the study period, corresponding to practice capacity and workflow differences.

**Table 1.** Practice characteristics and implementation of the rapid patient-reported outcome measure (RapidPRO).

Variable	Clinic 1	Clinic 2	Clinic 3	Clinic 4	Clinic 5	Clinic 6
Practice characteristics						
Number of new patients annually	—^a^	421	3000	992	1110	550
Patients with private insurance (%)	—	34	35	52	15	36
Patients with Medicare (%)	—	50	55	43	70	40
Total number of physicians	—	6	12	9	6	2
Total number of nurse practitioners	—	4	5	12	1	0
Total number of physician assistants	—	2	5	0	1	0
RapidPRO implementation characteristics						
PROMs^b^ (n=2252), n (%)	84 (3.7)	119 (5.3)	1411 (62.7)	428 (19)	79 (3.5)	131 (5.8)
Patients (n=695), n (%)	49 (7.1)	65 (9.4)	301 (43.3)	178 (25.6)	52 (7.5)	50 (7.2)
Date range	July 5, 2016 to June 6, 2017	February 14, 2017 to November 27, 2018	May 2, 2017 to December 25, 2018	July 11, 2017 to December 11, 2018	September 19, 2017 to January 30, 2018	January 30, 2018 to December 25, 2018
Weeks with PROMs, n (%)	31 (64.5)	54 (58)	55 (63.9)	72 (97.3)	11 (57.9)	45 (95.7)
Weeks without PROMs, n (%)	17 (35.4)	39 (41.9)	31 (36)	2 (2.7)	8 (42.1)	2 (4.3)
PROMs during weeks with PROMs (n), mean (SD)	1.71 (1.87)	1.27 (1.58)	16.22 (15.58)	5.71 (3.27)	3.95 (5.92)	2.73 (1.69)

a Missing data.

b PROM: patient-reported outcome measure.

A total of 2252 PROMs were completed by 695 patients across the 6 participating practices (Table 2). Of these 695 patients, 448 (64.5%) completed at least 2 PROMs, accounting for 69.1% (1557/2252) of the total PROMs. OAAs were reported in 92.8% (2089/2252) of the PROMs, and over half (1313/2252, 58%) of the PROMs were completed for 7 drugs. Cancer diagnosis was reported in only 580 PROMs, most commonly prostate (n=137, 23.6%), breast (n=111, 19.1%), colorectal (n=56, 9.7%), lung (n=35, 6%), ovarian (n=30, 5.2%), and pancreatic (n=21, 3.6%) cancer.

**Table 2.** Number of patient-reported outcome measures (PROMs) by medication.

Medication (patients, n)	PROMs, n	PROMS per patient, mean (SD)	PROMS per patient, median (IQR)	PROMS per patient (n), range
All (n=695)	2252	3.24 (3.18)	2 (1-4)	1‐39
Capecitabine (n=150)	372	2.48 (2.18)	2 (1-3)	1‐13
Palbociclib (n=52)	220	4.23 (3.62)	3 (1-6)	1‐15
Lenalidomide (n=41)	173	4.22 (3.76)	3 (2-6)	1‐15
Hydroxyurea (n=41)	168	4.10 (2.49)	4 (2-6)	1‐11
Abiraterone (n=59)	152	2.58 (1.93)	2 (1-3)	1‐10
Ibrutinib (n=43)	115	2.67 (1.61)	2 (1-4)	1‐7
Temozolomide (n=41)	113	2.76 (2.69)	2 (1-3)	1‐12

Among individuals completing at least 2 PROMs, the mean interval between assessments was 37 days (mean 37.54, SD 38.18 days, median 28 days) across all medications (Table 3). The mean and median were less than 30 days for capecitabine, palbociclib, lenalidomide, and temozolomide, consistent with their dosing cycles.

**Table 3.** Time between patient-reported outcome measures (PROMs) by medication.

Medication (patients, n)	PROMs, n	Time between PROMs (days), mean (SD)	Time between PROMs (days), median (IQR)	Time between PROMs (days), range
All (n=448)	1557	37.54 (38.18)	28 (14-42)	0‐342
Capecitabine (n=85)	222	29.57 (33.13)	21 (14-32)	0‐342
Palbociclib (n=37)	168	28.85 (19.23)	28 (15‐31.25)	1‐133
Lenalidomide (n=31)	132	27.28 (24.05)	24 (11-29)	0‐119
Hydroxyurea (n=35)	127	58.14 (47.81)	42 (28‐78.5)	3‐275
Abiraterone (n=37)	93	38.59 (37.41)	29 (14-55)	3‐247
Ibrutinib (n=29)	72	53.42 (52.14)	35 (14.75‐78.75)	3‐257
Temozolomide (n=23)	72	29.54 (26.91)	25.5 (8-35)	3‐147

### RapidPRO Clinical Metrics

#### Symptoms

**Figure 1.**
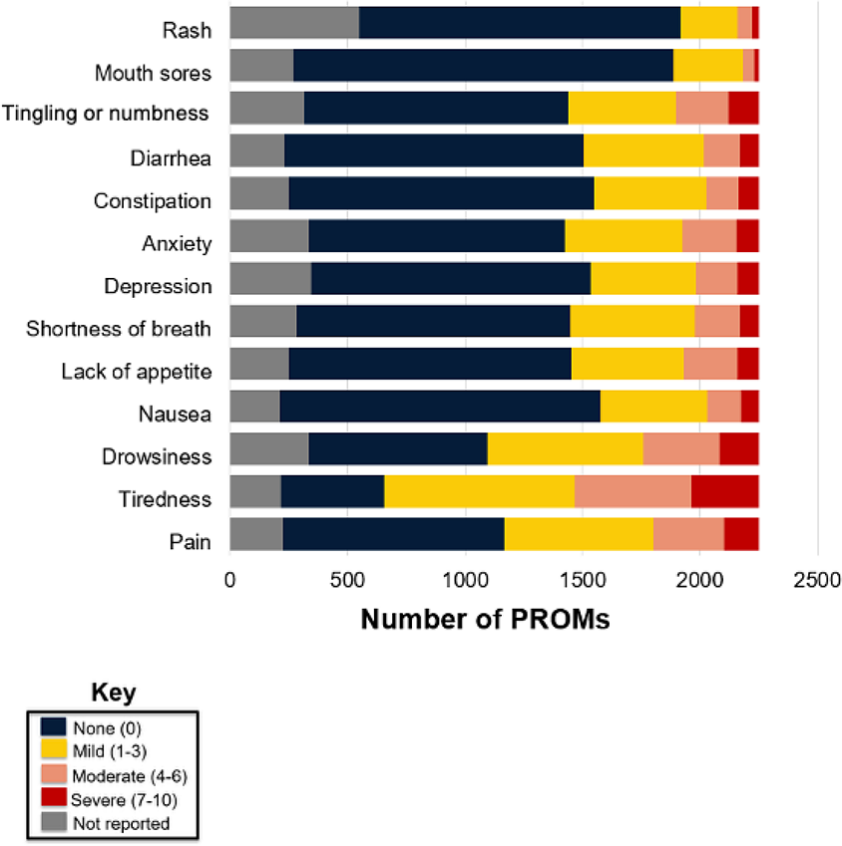
Edmonton Symptom Assessment System (ESAS) symptom severity in rapid patient-reported outcome.

Of the 2252 completed PROMs, 625 (27.8%) reported at least one severe symptom, and 1213 (53.9%) reported at least one moderate or severe symptom (Figure 1). A high symptom burden, defined as 4 or more total severe symptoms, was reported in 3.4% (n=76) of the PROMs. Additionally, 17.8% (n=400) of the PROMs reported at least 4 moderate or severe symptoms combined.

In addition to the symptoms reported in the ESAS, patients were asked to state their MBS, and patients could report more than one (Table 4). A total of 1045 MBSs were reported in 35.1% (790/2252) of the PROMs. Of the 695 patients, 323 (46.5%) reported an MBS. The most common MBS categories were constitutional including fatigue, gastrointestinal, musculoskeletal, and neurological (Table 4). MBSs reported were compared to the symptoms included in the RapidPRO, and ESAS symptoms accounted for 70% (731/1045) of the MBSs that were reported by patients. Fatigue was the most common severe and moderate ESAS symptom and the most commonly reported MBS. Among symptoms not covered by the ESAS, constitutional concerns were most common, and others occurred infrequently.

**Table 4.** Most bothersome symptoms reported in 790 patient-reported outcome measures by patients taking oral anticancer agents (N=1045).

Symptom category	Symptoms, n (%)
Constitutional symptoms (fatigue, fever, weakness, or weight loss)	323 (30.9)
Gastrointestinal	225 (21.5)
Musculoskeletal	101 (9.7)
Neurological	93 (8.9)
Integumentary or skin	83 (7.9)
Ears, nose, mouth, or throat	59 (5.6)
Cardiovascular	52 (5)
Psychiatric	41 (3.9)
Respiratory	18 (1.7)
Eyes	12 (1.1)
Genitourinary	11 (1.1)
Hematologic or lymphatic	8 (0.8)
Allergic or immunologic	2 (0.2)
Endocrine	1 (0.1)
Financial	1 (0.1)
Other	15 (1.4)

#### Confidence

Overall, most patients expressed high confidence in both managing and seeking help for OAA-related symptoms. Patients reported their confidence to self-manage their symptoms related to OAAs in 72.9% (1642/2252) of the completed PROMs (Table 5). Of those 1642 PROMs, 1252 (76.2%) rated their confidence as high (7-10). Confidence to seek medical care for symptoms related to OAAs was reported in 74.8% (1685/2252) of the PROMs. Of those 1685 PROMs, 1475 (87.5%) reported confidence as high.

**Table 5.** Confidence, adherence, and reasons for nonadherence reported in the rapid patient-reported outcome measure (PROM; N=2252).

Outcome	PROMs, n (%)
Confidence to seek medical care (n=1642)	
Low (0‐3)	236 (14.4)
Moderate (4-6)	154 (9.4)
High (7-10)	1252 (76.2)
Confidence to self-manage symptoms (n=1685)	
Low (0‐3)	119 (7.1)
Moderate (4-6)	91 (5.4)
High (7-10)	1475 (87.5)
Self-rated adherence to oral anticancer agents (n=1705)	
Excellent	1220 (71.6)
Nonadherent or less than excellent	485 (28.4)
Reasons for nonadherence (n=430)^a^	
“I did not have money to pay for this medicine.”	24 (5.6)
“I did not have the medicine because the pharmacy was out of this medicine.”	10 (2.3)
“I experienced side effects from this medicine.”	156 (36.3)
“I have concerns about possible side effects.”	83 (19.3)
“I have concerns about long term effects.”	64 (14.9)
“I do not think I need this medication anymore.”	9 (2.1)
“I do not think that this medication is working for me.”	14 (3.3)
“I have trouble managing all the medicines I take.”	13 (3)
“I would have taken it but simply missed it.”	125 (29.1)
“I would have taken it but missed it because of a busy schedule.”	42 (9.8)
“I would have taken it but have problems forgetting things in my daily life.”	43 (10)
Other	109 (25.3)

a Patients could select more than one reason.

#### Adherence

The medication adherence assessment was completed in 75.7% (1705/2252) of the total PROMs, and 28% (485/1705) of PROMs had a rating defined as nonadherent (Table 5). Commonly reported reasons for medication nonadherence included side effects or concerns about side or long-term effects (303/2252, 13.5%) and forgetfulness or missed doses (223/2252, 9.9%).

#### Relationships Among Clinical Metrics

**Figure 2.**
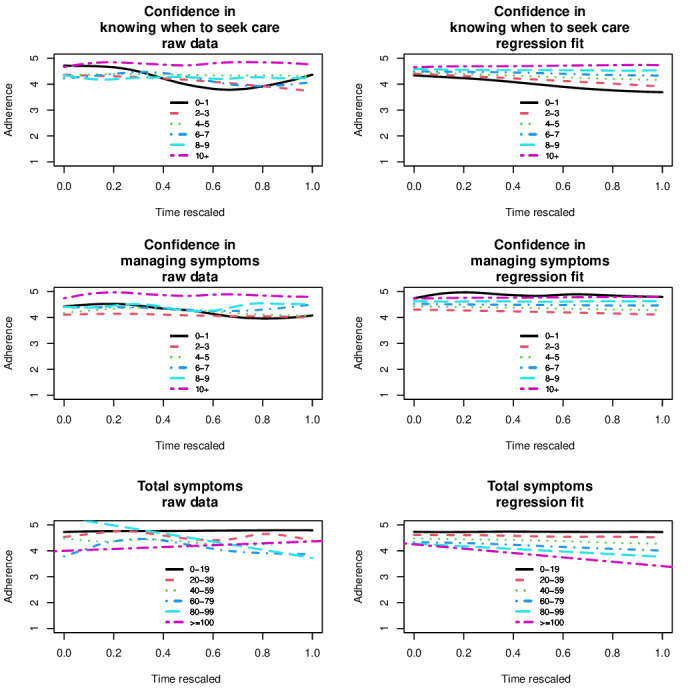
Variation in patient-reported outcome measures (PROM) of total symptoms (sum of symptoms 0 to >100 [worst]), confidence to manage symptoms, confidence to know when to seek care (0 to 10 [best]), and adherence (1 to 5 [best]) among patients with multiple PROMs using a predicted mixed interaction model (left) and a smoothing spline for each line showing raw data (right).

The relationships among symptom burden, confidence, and medication adherence were examined among individuals completing at least 2 PROMs (Figure 2). In the figures developed from the predictive mixed interaction models (left panels in Figure 2), more symptoms and less confidence were related to poorer medication adherence over time. In the raw data (right panels in Figure 2), these relationships were more variable, although higher total symptom scores and lower confidence levels showed similar trends.

In the mixed-effects models for patients with multiple PROMs, those with low confidence to self-manage symptoms or low confidence to seek medical attention were also more likely to report medication nonadherence (*P*<.001 and *P=*.001, respectively). Individuals with PROMs that reported a moderate or severe symptom on the ESAS were more likely to be nonadherent to OAA therapy (*P*=.02), as were patients who were receiving capecitabine (*P*=.005).

## Discussion

### Principal Findings

Using PROMs to evaluate symptom burden and medication adherence in patients receiving OAAs should be considered to enhance the quality of care as the responsibility for managing symptoms and medication adherence lies heavily on patients [32,43]. A short, easy-to-use PROM was used in 6 clinical practices, and it was completed by almost 700 patients. Importantly, it can be completed by patients in approximately 3 minutes. It comprises previously validated survey items that assess symptom burden, patient self-management confidence, and medication adherence concisely for all OAAs and allows oncologists and oncology professionals to respond in a timely way [27,32,34,35,38,39,44]. This PROM can be used broadly for all OAAs as an initial screening tool vs the need to customize for each drug or cancer type. This is helpful when establishing a consistent workflow within practices, especially given the large number of OAAs that are available. The practices did show variability in the number of completed PROMs each week during their participation, as well as variation in administration mode.

Data from the RapidPRO were used to explore factors associated with medication nonadherence, and individuals with more severe symptoms and lower confidence in self-management of symptoms were more likely to be nonadherent to therapy. These findings are consistent with those of previous studies. Patients have previously indicated experience or fear of side effects as a main reason for medication nonadherence [5,16,27,45-48]. In addition, patients with colorectal cancer who experienced more severe or episodic side effects have expressed more difficulty with self-management, which led to medication nonadherence [27,49,50]. Patients with lower confidence in self-managing symptoms may also be unsure of whether symptoms are expected or require clinical attention, which can lead to skipped doses and medication nonadherence. Prior studies of oral oncolytics show that lower self-efficacy is associated with difficulty performing self-management behaviors and poorer adherence [27]. Difficulty managing symptoms can also heighten distress and impair daily functioning, further disrupting medication-taking routines [28]. Inadequately managed symptoms can also signal early clinical deterioration and are associated with greater unplanned health care use [29]. These findings suggest that confidence ratings on the RapidPRO may function as an early indicator of patients who may benefit from proactive symptom management interventions and adherence support.

A question was added to the PROM related to the patients’ MBS given the varying medication side effect profiles between OAAs in the event that patients were experiencing symptoms that were not included in the ESAS. We found that, even with this additional reporting option, the ESAS captured most symptoms that patients experienced, further justifying its use within RapidPRO [19,33,36].

RapidPRO provides a brief, clinically focused alternative suitable for routine use with patients receiving OAAs compared with other cancer patient-reported outcome tools such as the Functional Assessment of Cancer Therapy–General (27 items) and the European Organisation for Research and Treatment of Cancer Quality of Life Questionnaire Core 30 (30 items), which assess broad physical, emotional, social, and functional domains and require more time to complete [51]. Patient-Reported Outcomes Measurement Information System measures offer flexible item banks for general health-related functioning and include a generic medication adherence scale, but they are not cancer specific and do not capture symptom burden or confidence in managing OAA-related side effects [52]. RapidPRO instead integrates validated ESAS symptom items with patient confidence and medication adherence—domains directly relevant to daily OAA self-management. Consistent with recent reviews showing that short, symptom-focused tools are more feasible in busy oncology workflows than comprehensive health-related quality of life instruments [53], RapidPRO fills a distinct niche by delivering rapid, actionable information to guide clinical care.

Given the extent of symptom experience and medication nonadherence, these results suggest the critical need to use a process that routinely assesses toxicities, medication adherence, and confidence, and RapidPRO is not overly burdensome or complex. By identifying patients who are at risk of medication nonadherence, practices have the ability to provide additional medication teaching, symptom management support, and other targeted interventions. Implementing a PROM into daily oncology workflows remains challenging. Despite standardized data collection procedures across all clinics, the number of completed RapidPRO assessments varied due to differences in staffing and workflow capacity. This represents typical variation in how PROMs are incorporated into routine care in community oncology settings. The oncology practices experienced common barriers, including staffing constraints, visit schedule variability, and differences in administration processes. These challenges reflect prior evidence showing that PROM implementation is often hindered by time burden, workflow disruption, and limited technical integration [54]. Cancer-specific implementation studies similarly highlight workflow variability as a key barrier to sustaining routine PROM collection [55].

In this project, the MOQC helped mitigate these barriers by providing onboarding training, a written user guide with follow-up thresholds, ongoing support, and flexible administration options (paper or telephone). These strategies facilitated the integration of RapidPRO into routine care, although further workflow standardization may improve consistent use. Moreover, recent evidence shows that PROMs improve mortality and health-related quality of life at 12 weeks with moderate certainty [30,56]. Collectively, these results provide strong external evidence that patients would benefit from PROM assessments such as RapidPRO. This single instrument could be used for all OAAs. The introduction of new OAAs also with toxicities or side effects provides additional impetus.

One of the limitations of this study is that, because it focused on testing the feasibility of using RapidPRO in clinical practice, only information included in the PROM itself was collected. Detailed clinical characteristics from electronic medical records such as cancer stage, time since diagnosis, or treatment intent were not captured, and cancer diagnoses were missing for many PROMs. While these data reflect real-world clinical use, they limit the ability to compare results across clinical subgroups. Future work linking RapidPRO data with electronic medical record information will be important for evaluating how RapidPRO performs across specific cancers and treatment pathways.

### Conclusions

An easy-to-administer RapidPRO for monitoring patients taking OAAs was used across 6 sites, with variability in its daily use. Symptom burden was common for patients taking OAAs, with 17.8% (400/2252) of the PROMs reporting at least 4 moderate or severe symptoms combined, and 28% (485/1705) of the PROMs reported less than excellent medication adherence. The most prevalent patient-reported reason for medication nonadherence was side effects. By implementing the RapidPRO, practices can identify patients who experience symptoms, as well as those who report medication nonadherence.

## Supplementary material

10.2196/85201 Multimedia Appendix 1 Michigan Oncology Quality Consortium rapid patient-reported outcome measure resource links.

## Abbreviations

ESAS: Edmonton Symptom Assessment System
HIPAA: Health Insurance Portability and Accountability Act
MBS: most bothersome symptom
MOQC: Michigan Oncology Quality Consortium
OAA: oral anticancer agent
PROM: patient-reported outcome measure
RapidPRO: rapid patient-reported outcome measure
REDCap: Research Electronic Data Capture
