# Supplementary material for: A Short Patient-Reported Outcome Measure for Oral Anticancer Agents: Multicenter Observational Study
Source: JMIR Form Res. 2026 Feb 18;10:e85201. doi: 10.2196/85201 (PMC12916090; doi:10.2196/85201)
Supplement: Multimedia Appendix 1 [file formative-v10-e85201-s001.docx]

**Multimedia Appendix**

**MOQC RapidPRO Resource Links**

This is a Multimedia Appendix to a manuscript submitted to JMIR Formative Research. The following publicly available resources from the Michigan Oncology Quality Consortium (MOQC) provide detailed information on the RapidPRO tools used in this study:

- Patient-Reported Outcome (PRO) Intake Document
  Available at: <https://moqc.org/resources/oral-oncolytics/>
- Patient-Reported Outcome (PRO) Assessment *RapidPRO* (English , Chinese , Arabic, Spanish)
  Available at: <https://moqc.org/resources/oral-oncolytics/>
- Patient-Reported Outcome (PRO) Tool Guide
  Available at: <https://moqc.org/resources/oral-oncolytics/>
- Oral Oncolytic Monitoring Note Template
  Available at: <https://moqc.org/resources/oral-oncolytics/>
